# Supermarket Healthy Eating for Life (SHELf): protocol of a randomised controlled trial promoting healthy food and beverage consumption through price reduction and skill-building strategies

**DOI:** 10.1186/1471-2458-11-715

**Published:** 2011-09-22

**Authors:** Kylie Ball, Sarah A McNaughton, Cliona Ni Mhurchu, Nick Andrianopoulos, Victoria Inglis, Briohny McNeilly, Ha ND Le, Deborah Leslie, Christina Pollard, David Crawford

**Affiliations:** 1 Centre for Physical Activity and Nutrition Research, Deakin University, Burwood Hwy, Burwood, 3125, Australia; 2 Clinical Trials Research Unit, University of Auckland, Auckland, New Zealand; 3 Coles Supermarkets, Toorak Rd, Hawthorn East, 3123, Australia; 4 Deakin Health Economics, Deakin University, Burwood Hwy, Burwood, 3125, Australia; 5 School of Public Health & Preventive Medicine, Monash University, Melbourne, 3004, Australia; 6 Nutrition & Physical Activity Branch, Health Department Western Australia, East Perth 6004, Australia

## Abstract

**Background:**

In the context of rising food prices, there is a need for evidence on the most effective approaches for promoting healthy eating. Individually-targeted behavioural interventions for increasing food-related skills show promise, but are unlikely to be effective in the absence of structural supports. Fiscal policies have been advocated as a means of promoting healthy eating and reducing obesity and nutrition-related disease, but there is little empirical evidence of their effectiveness. This paper describes the Supermarket Healthy Eating for LiFe (SHELf) study, a randomised controlled trial to investigate effectiveness and cost-effectiveness of a tailored skill-building intervention and a price reduction intervention, separately and in combination, against a control condition for promoting purchase and consumption of healthy foods and beverages in women from high and low socioeconomic groups.

**Methods/design:**

SHELf comprises a randomised controlled trial design, with participants randomised to receive either (1) a skill-building intervention; (2) price reductions on fruits, vegetables and low-joule soft drink beverages and water; (3) a combination of skill-building and price reductions; or (4) a control condition. Five hundred women from high and low socioeconomic areas will be recruited through a store loyalty card program and local media. Randomisation will occur on receipt of informed consent and baseline questionnaire. An economic evaluation from a societal perspective using a cost-consequences approach will compare the costs and outcomes between intervention and control groups.

**Discussion:**

This study will build on a pivotal partnership with a major national supermarket chain and the Heart Foundation to investigate the effectiveness of intervention strategies aimed at increasing women's purchasing and consumption of fruits and vegetables and decreased purchasing and consumption of sugar-sweetened beverages. It will be among the first internationally to examine the effects of two promising approaches - skill-building and price reductions - on diet amongst women.

**Trial Registration:**

Current Controlled Trials ISRCTN39432901

## Background

Despite the well-established benefits of good nutrition for health, large proportions of the population in many countries do not consume the types or amounts of foods and drinks that are important for leading healthy lives [1]. For instance, more than 80% of Australian adults do not eat the recommended amount of vegetables, and over 40% do not eat enough fruit for good health [2]. High intakes of sugar-sweetened beverages are also a key contributor to obesity risk and associated adverse health outcomes [3,4]. There is, therefore, strong impetus for promoting increased intakes of fruits and vegetables, and decreasing intakes of sugar-sweetened beverages in the population. However, the most effective strategies for doing so remain unknown.

Initiatives aimed at improving population diet can involve 'downstream', individually-targeted approaches, or 'upstream' structural approaches. There are a number of important reasons to examine the effectiveness of individually-targeted ('downstream') interventions for improving diet. For example, our previous work has demonstrated that the strongest correlates of women's fruit and vegetable intakes were not upstream structural factors, but rather intrapersonal factors such as nutrition knowledge and health considerations [5]. Observational studies have identified a number of other potential intrapersonal determinants of eating behaviours, including confidence or skills in meal planning/preparation/cooking, and perceived financial costs of healthy eating [6,7]. Difficulty with budgeting is often reported as a key barrier to healthy eating [8,9]. Collectively, such findings suggest that improving individuals' skills in planning, budgeting for, procuring and preparing healthy foods may be important goals of healthy eating interventions.

Increasingly, however, it is recognised that traditional 'education'-based nutrition promotion strategies that rely solely on individual responsibility are unlikely to be effective in the absence of broader structural supports. Fiscal policies, such as taxations or subsidies for certain foods or beverages, represent one such structural support that has recently received considerable attention. Recent global increases in the costs of foods, by as much as 75% in recent years [10], attributable to factors such as drought, rising oil prices, increased demand for certain crops such as corn for biofuel production, and declining world-food stockpiles [11], have placed increased food-related financial strain on individuals across a range of socioeconomic circumstances. There is strong evidence that prices influence food consumption choices [12]. 'Upstream' fiscal intervention approaches such as reducing the prices of healthier foods in relation to less healthy alternatives are hence potentially valuable strategies for promoting healthier eating amongst large sectors of the population. In Australia and internationally, fiscal food policies have been advocated as a means of promoting healthy eating and reducing obesity and associated health outcomes [1,13,14] but there remains little empirical evidence of their effectiveness in populations [15].

### Previous skill-based and price reduction nutrition interventions

While there is now a body of observational data indicating the likely influences on eating behaviours, there remains a paucity of robust intervention research about the most effective means of changing behaviours and promoting healthy eating. This is particularly the case amongst persons experiencing socioeconomic disadvantage, who are at high risk of nutrition-related diseases [16-18]. The existing evidence on the effectiveness of skills-based or price reduction approaches to improving diet has focused primarily on fruit and vegetable consumption. Two reviews have reported on the effectiveness of different behavioural approaches, including skill-building, in increasing fruit and vegetable intakes amongst adults [19,20]. Those reviews highlighted that behavioural interventions show promise in increasing the quantity and/or variety of fruit and vegetable intakes. They also identified a number of common elements to effective interventions, including goal-setting, providing skills to achieve goals, provision of recipes and motivational newsletters. Printed information appeared to be an effective, more feasible and less expensive alternative to face-to-face or telephone contact. An important gap identified in the reviews was the fact that no studies had at that stage been conducted outside of the United States or Europe; and few provided evidence for the efficacy of interventions in low-income or socioeconomically disadvantaged individuals.

In the Australian context, one skill-based nutrition promotion program, 'Food Cent$', trialled an innovative intervention approach aimed primarily at increasing food budgeting skills to support people with limited budgets to allocate money to healthier foods [21]. The intervention emphasised value for money by comparing foods on a cost per kilogram basis and provided resources for participants to develop budgeting, cooking and shopping skills. While this program showed positive changes in cooking, shopping and eating behaviours, all outcome measures were self-reported, and there was no control group, which limited conclusions about the intervention's effectiveness.

In terms of existing price reduction approaches, one review [12] of price-related nutrition interventions concluded that while price reduction strategies show considerable promise as effective approaches to promoting healthy eating, most of the existing research has focused on relatively contained settings, such as schools or worksites, and there is a need for further research on the effectiveness of such strategies in the broader community, such as through supermarkets. There is also a need to consider the effect of such fiscal strategies relative to, and in tandem with, other promising approaches, such as skill-building strategies.

Only one previous published study, the SHOP trial in New Zealand [22], has investigated the effectiveness of individually-targeted nutrition education in conjunction with price reduction strategies in promoting healthy eating in a real-world setting, using a randomised controlled trial design. That study found a significant and sustained effect of price discounts on food purchasing, but no impact of the education strategies on food purchasing or nutrient intakes. However, the education component employed in that study primarily comprised tailored messages suggesting substitution of unhealthy foods with specific healthier products. It was not strongly based on formal behaviour change theories or strategies, and although it did provide recipes, it did not address food budgeting, purchasing, or preparation skills. In addition, the SHOP study population was generally well-educated, and generalizability to a lower-educated or more disadvantaged population is unknown.

Notably, none of the above reviews or studies reported on the mediators or mechanisms of dietary change resulting from interventions. An understanding of these mediators is important for highlighting the most successful intervention elements and how they operate to change behaviour. Similarly, there remains very little evidence on the cost-effectiveness of intervention approaches to promoting healthy eating. With limited resources available for public health, it is increasingly important to understand the most effective specific intervention components, their combination effects, and the 'real world' implementation opportunities to establish whether interventions would represent good 'value-for-money'

This paper describes the protocol for the Supermarket Healthy Eating for Life (SHELf) study, a randomised controlled trial that will build on an important intersectoral partnership with Coles Supermarkets, a major national supermarket chain, and the National Heart Foundation of Australia. Supermarkets are a major controller of food access, pricing and affordability [23]. Supermarkets and grocery stores accounted for 64% ($75 billion) of the total food retail in Australia in 2007-8 [23]. Coles Supermarkets are the second-largest grocery chain in Australia, with around 740 stores nationally. Coles Supermarkets have a store loyalty program called FlyBuys. Shoppers who sign up to FlyBuys are given a credit card style membership card which can be scanned every time a purchase above five Australian dollars is made at a participating FlyBuys business. This allows members to collect points which can then be exchanged for rewards. The National Heart Foundation of Australia is a not-for-profit non-government organization whose mission is to reduce suffering and death from heart, stroke and blood vessel disease in Australia, with nutrition promotion a key focus.

SHELf aims to address the gaps in the existing literature identified above. It tests the effectiveness and cost-effectiveness of a skill-building intervention, a price reduction intervention, and a combined skill-building and price reduction intervention, against a control condition, in promoting purchasing of fruits and vegetables, reducing purchasing of sugar-sweetened soft drinks, and increasing purchasing of low-joule soft drinks/water amongst women (of both high and low socioeconomic status). Secondary aims are to test the impact of the intervention on increasing self-efficacy for, and perceived affordability of, healthy eating, and to examine the contribution of self-efficacy and perceived affordability as mediators of changes in purchasing and consumption behaviours resulting from the intervention. The SHELf study will build upon and extend the SHOP study by drawing on two theoretical frameworks (Social Ecology Theory [24] and Social Cognitive Theory [25]), and by incorporating a skills-based intervention component (rather than education alone), utilizing strategies shown to be feasible and effective in the Australian context in the Food Cent$ study.

The study tests the key null hypotheses that, at the end of the three-month intervention, and at the six-month follow-up, there will be no differences in:

• fruit and vegetable purchasing or consumption;

• purchasing or consumption of sugar-sweetened high-joule soft drinks versus low-joule soft-drinks/water;

• the proposed mediators, self-efficacy and perceived affordability of healthy eating;

• the costs to society,

between the skill-building intervention participants and the controls; the price reduction intervention participants and the controls; or the combined skill-building and price reduction intervention participants and controls.

## Methods/design

### Study design and setting

The Supermarket Healthy Eating for Life (SHELf) study is a randomised controlled trial, conducted amongst women selected from one relatively advantaged, and one relatively disadvantaged, neighbourhood, selected according to the Australian Bureau of Statistics' Socioeconomic Index for Areas (SEIFA) index of relative advantage/disadvantage [26]. This SEIFA index is an area-based indicator of the socioeconomic conditions of people living in an area, based upon aggregated social and economic information from the population Census (such as the proportion of low-income households, or of people with a tertiary education). Women will be randomised after recruitment and baseline measurement to one of four intervention arms: (1) skill-building; (2) price reductions on fruits, vegetables and low-joule soft drink beverages and water; (3) a combination of skill-building and price reductions; or (4) a control group. Ethical approval has been granted for the study by Deakin University's Human Research Ethics Committee (HEAG-H 12/10). The study is funded by the Australian National Health and Medical Research Council (ID594767).

**Figure 1:**
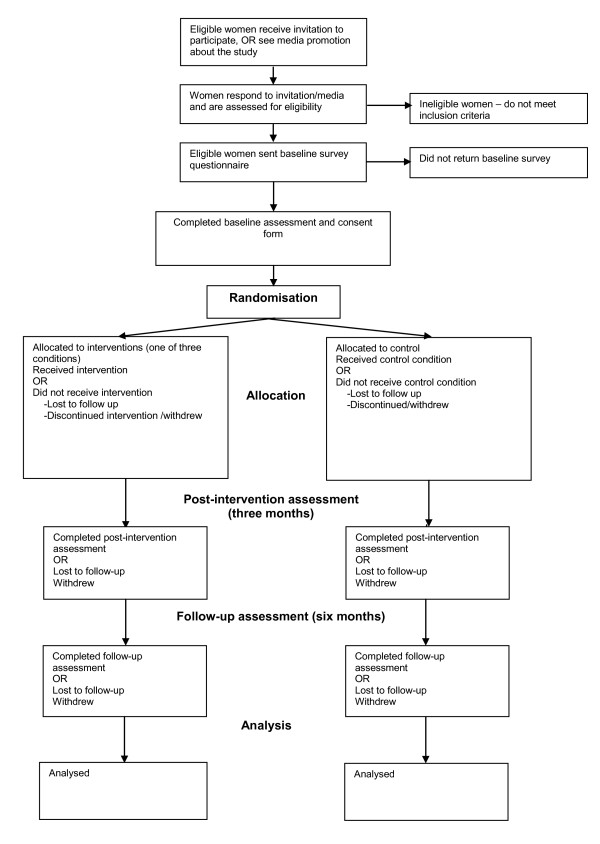
**SHELf study design**.

This study will employ a three-month intervention and pre-, post- and six-month follow-up assessments of intervention effects, as detailed in Figure 1. It will focus on women who shop at Coles Supermarkets in the targeted areas in metropolitan Melbourne.

### Participants

Women will be targeted in this study as they are most often responsible for food choice, purchasing and preparation, particularly in family households [27,28]; they, as the gatekeepers, often influence the amount and type of food eaten by other family members.

#### Participant identification

Two Coles Supermarket stores, one in an advantaged and one in a disadvantaged neighbourhood, were purposively chosen in order to sample participants from both high and low socioeconomic backgrounds. The stores were also selected for pragmatic reasons based on their proximity to Deakin University (less than 25 km) and the number of women who spoke English as a proportion of the total number of women living in the neighbourhood (according to the ABS Community Profiles derived from the 2006 Australian Census) [29]. These stores were located in Melbourne metropolitan suburbs of Hawthorn East and Reservoir. Using Geographic Information Systems software, two catchments were identified, each with a 5 km radius from the initially selected store.

Potential participants will be contacted via a mail-out of a study recruitment pack to women living and shopping in the target catchments. This mail out will be specifically targeted to Coles customers who are members of the Coles loyalty program FlyBuys. The recruitment pack will contain a letter from the FlyBuys team, who operate the Coles store loyalty card; a recruitment brochure and a reply-paid envelope. Targeting will be done by employees at Coles and Loyalty Pacific Pty Ltd (manager of the 'FlyBuys' program) using their sales and customer databases respectively. To maintain the privacy of FlyBuys members, the final mail out will be co-ordinated by Loyalty Pacific Pty Ltd. Women will be eligible to receive the recruitment pack if they are aged 18-60 years, participate in the FlyBuys program and are a regular shopper at any of the Coles stores in either of the catchments (regular being defined as shopping on at least six days in a 12-week period, or approximately once every two weeks or more). A media release targeting local newspapers in the study catchment areas will also be used as a recruitment strategy; women responding will be invited to participate if they satisfy eligibility criteria, and have, or are willing to obtain, a FlyBuys card. Compensations (shopping vouchers to the value of $60.00 and store loyalty points to the value of $15.00) will be offered to all participants. All women recruited into the study will be asked to provide the identifying number for their existing store loyalty (FlyBuys) card or will be asked to apply for a card if they do not already use one, so that fruit, vegetable and beverage purchases pre-, during and post-intervention can be obtained through accessing electronic sales data linked with the loyalty cards.

#### Eligibility criteria

Women will be screened for eligibility either via the recruitment brochure or telephone screening using the following inclusion criteria: they must be a woman, aged 18-60 years; the main household food shopper; shop regularly at Coles supermarkets in one of the two defined catchment areas; willing to use their FlyBuys customer loyalty card at Coles supermarkets in the next nine months; able to give written informed consent to participate in the study; willing to give information about total household income; willing to have purchase data collected and analysed; able to speak, read and write in English; and the only woman in their household taking part in the study.

#### Sample Size

Sample size calculations are based on the ability to detect increases in fruit and vegetable purchasing of at least 0.5 serves per day (in Australia, a standard serve is equivalent to 75 grams vegetables or 150 grams fruit). There is evidence that this increase is feasible; for example, a review of the literature on fruit and vegetable intervention studies [19] found an average increase of 0.6 serves of fruit/vegetables per day across studies. The Australian Go for 2&5 campaign achieved a population net increase of 0.8 in the mean number of servings of fruit and vegetables per day over three years [30]. Even small population increases in fruit and vegetable consumption are meaningful. For example, an increase of 80 g per day of fruit and vegetables has been estimated to reduce the risk of ischaemic heart disease by 10%, ischaemic stroke by 6%, lung cancer by 4% and oesophageal cancer by 6% [31].

Of the outcome variables (fruit and vegetable purchasing), it is estimated that the most challenging to shift will be vegetable purchasing, therefore we have based our sample estimates on this outcome. There are no recent national dietary survey data in Australia (with the last national survey of adults conducted in 1995), so we have based our estimates on more recent data collected in a large community-based survey of over 1500 women [5]. In that study, women of low socioeconomic position reported consuming on average 1.9 serves of vegetables per day (0.5 serves fewer than those of higher position), with a standard deviation of 1.1. Therefore, to detect an increase of 0.5 serves of vegetables, using the following formula to calculate the sample size for a continuous measure:

N per group = 2 * SD^2 ^* (Zsign + Zpower)^2 ^/delta^2 ^, where SD = 1.1; delta = 0.5; Zsign for 5% type 1 error is 1.96 and Zpower for 20% type 2 error is 0.84, N per group = 76, totalling 304. Inflating our estimate to adjust for attrition/loss to follow-up (conservatively estimated at around 10% at each measurement wave), and to account for potential design effects based on sampling within catchment areas (conservatively estimated at 1.1 or an inflation of 10%), our total minimum sample size is 304/0.70 * 1.1 = 478 (rounded up to 500, or 125 in each group).

A sample of this size is also sufficient to examine mediation effects of at least medium size using the McKinnon approach [32]. We will recruit both participants and controls from each of the two catchment areas (catchment area will be controlled for in analysis).

#### Randomisation

Eligible women will be asked to complete baseline survey measures before being randomly allocated to one of the four study conditions: skill-building, price reduction, skill building and price reduction or control. The randomisation will be undertaken using a computer-generated blocked randomisation sequence, stratified by low and high socioeconomic status of the catchment area from which women were sampled, and will be produced by the study statistician, with treatment allocation not available to investigators at any stage.

#### Blinding

Because of the nature of the intervention, by which intervention participants receive either skill-building materials, price reductions or both, study participants cannot be blinded to intervention arm. Research staff administering the intervention will also not be blinded to the intervention, as they will be required to liaise/communicate with participants through the course of the intervention; research staff however will not be involved in the randomisation process, and they will not have access to the randomisation sequence used. The statistician involved in creating the randomisation sequence will have no contact with participants or their information. The chief investigators and the data manager, who will be involved in data preparation and analysis, will also be blinded to intervention arm, and will have no contact with participants at any time during the intervention.

### Intervention approach

#### Theoretical framework

The intervention will be informed by Social Ecology Theory [24], which proposes that behaviour is influenced by ongoing transactions between the individual and the environment, and that efforts to promote health should integrate both behavioural (e.g., goal-setting, skill-building) and environmental (e.g., price reductions) health promotion strategies. It will also be guided by Social Cognitive Theory [25], which proposes that individuals adopt new behaviours through social learning, either through imitation of others, or through media sources, as proposed here. According to social cognitive theory, effective modelling provides individuals with skills and strategies for adopting and maintaining a behaviour in different circumstances. Individuals are more likely to adopt and maintain a behaviour if they value the outcome of the new behaviour, and believe themselves to be capable of undertaking it (high self-efficacy). In addition, the intervention will draw on empirical evidence of the key determinants of eating behaviours, including our own past work suggesting the importance of addressing nutrition knowledge, budgeting and food planning and preparation skills, perceived and actual foods costs, and other barriers.

#### Skill building intervention

An intervention mapping approach was undertaken, consistent with the recommendations of Bartholemew et al. [33] to ensure that the intervention was based on a strong theoretical, empirical, and practical foundation. As part of the mapping approach, matrices of change objectives based on the determinants of eating behaviours were developed. Intervention material content was then sourced, via a literature and hand search of existing nutrition promotion materials that target increasing fruit and vegetable consumption and healthy beverage consumption, with efforts made to include materials targeting socioeconomically disadvantaged individuals. New materials were developed where there was insufficient or inadequate existing content to address the study aims. The findings of the search and mapping process identified that printed materials and some social support/interactive components would be effective strategies to address the study aims, and there was a need for a particular focus on budgeting. As a result, a set of eight skill-building newsletters and accompanying behaviour change and supplementary resources (including activities such as budgeting worksheets, goal-setting and self-monitoring exercises, and two recipes as well as links to further online recipes and resources), were developed, along with a complementary online forum to provide interaction both amongst intervention participants, and with an expert (Accredited Practising Dietitian). Separate forums have been created for women in the separate skill-building, and skill-building plus price reduction intervention arms, to avoid contamination across groups.

Participants in the skill-building intervention groups will be provided with the printed newsletters and supplementary materials, which will be mailed every week for the first four weeks and then every two weeks for the remaining duration of the three-month intervention. This component will be tailored to women with and without children (whether or not they reported caring for children under the age of 12 years). Access to the online forum will be available to participants for the whole three months. Elements of the intervention to be provided through print and online media will include:

##### Education

Awareness-raising of the importance of fruit and vegetables to healthy eating and health in general, as well as the range of low-cost fruits and vegetables available and the relative costs compared with other foods. In-season produce will also be identified. This component is aimed at increasing the proposed theoretical mediators, perceived knowledge and value of increased fruit and vegetable consumption and perceived affordability of fruits and vegetables.

##### Skill-building

To foster behavioural skills in budgeting; meal planning; and meal preparation strategies including provision of shopping lists linked to simple recipes. This is aimed at increasing the theoretical mediators, self-efficacy and perceived affordability.

##### Goal-setting

This is a key behaviour change activity identified as a common element of successful previous fruit/vegetable interventions. For example, women will be encouraged to increase their and their families' vegetable consumption to meet the Australian recommendation of five 75 gram serves/day.

##### Overcoming barriers

Tips will be provided on overcoming commonly-reported barriers to increased fruit and vegetable consumption identified from our previous work. For example, suggestions will be provided on engaging familial involvement/support, given theoretical arguments and previous literature on the importance of social influences on behaviour. This component targets the theoretical mediator of perceived barriers. In addition, the newsletters and online forum will be tailored according to whether or not women have young children living with them, since the presence of young children (under age 12) has been associated with particular barriers to healthy eating behaviours [34].

##### Social support

Access to the online forum will enable women to share ideas and support each other, as well as benefit from the reinforcement of messages provided in the newsletters. The forum will contain discussion boards with threads that coincide with the newsletter education and skill-building content. An Accredited Practicing Dietitian will answer any questions and add posts regularly to prompt discussion. For women with children, newsletters will include tips on garnering children's support/engagement in the healthy eating behaviour change processes.

A summary of the newsletter content, including the target mediators, the corresponding newsletter communication objectives, and the barriers and enablers targeted in each newsletter, are shown in Table 1.

**Table 1:** Summary of skill-building intervention newsletter objectives, targeted mediators and content

**Newsletter Week (no)**.	Objective/target	Primary communication objective	Secondary objective (barriers and promoters addressed)
1(1)	Knowledge	Why you should eat more fruit and vegetables and drink water	- Health benefits - How much to eat/drink - Set goals to increase amount of vegetables

2(2)	Self-efficacy for healthy eating, perceived affordability of fruit & vegetables	Planning for healthy eating	- Planning a menu (inventory of items on hand) and writing a shopping list - Planning to drink more water

3(3)	Self-efficacy for healthy eating	Shop smart	- Saving money at the supermarket (using canned or frozen when fruit & vegetables not in season) - Understanding labels - Setting goals (related to buying more fruit & veg while shopping)

4(4)	Perceived affordability of fruit & vegetables, self-efficacy for healthy eating	Confidence in the kitchen	- Creating quick simple meals from items on hand (include cooking methods, adding fruit & vegetables to simple meals) - Preventing wastage - Fruit juice and sugar

5(5)	Perceived barriers	Saving time and money	- Meal ideas for saving time - Food safety (to avoid waste) - Soft drinks and sugar

7(6)	Perceived barriers	Trying different fruit & vegetables	- Tasting new/different fruit & veg - Healthy eating outside the home - Trying sweet-drink alternatives

9(7)	Self-efficacy for healthy eating, perceived barriers	Confidence in eating healthy at all times	- Modifying recipes - Eating fruit & vegetables in social situations - Energy deficit when reducing consumption of sweetened drinks

11(8)	Self-efficacy for healthy eating, perceived barriers, awareness	Revision	- Resources to use for continuing to eat healthy - Revision of key messages

Two sets of newsletters were created, tailored to women with, and to women without children aged 12 years or younger. All versions have been pilot tested in a sample of women from a range of socioeconomic backgrounds, and were modified on the basis of pilot feedback on the formatting, content and language (for example, in response to repeated requests for more recipes, the number of recipes accompanying newsletters was doubled).

#### Price reduction intervention

Participants in this intervention arm will receive price discounts equivalent to 20%, which will be applied electronically at the supermarket checkout on all healthier options (all fruits and vegetables, including fresh, tinned and frozen, and diet- or low-calorie soft drinks or water) purchased by study participants. A reduction of this magnitude is considered appropriate, since it is unlikely to lead to the unintended consequence of promoting additional snack food items that a larger price reduction might encourage [12]. The focus on low-joule soft drinks is justified from an energy-balance/obesity prevention perspective, as a substitute for their more commonly-consumed, high-joule sugar-sweetened versions, which is consistent with the Dietary Guidelines for Australian Adults [35]. Discounts will be linked to participants' store loyalty (Fly-Buys) cards, to ensure they receive a discount upon swiping their card at checkout. Participants will be provided with a list of all items for which price reductions apply at the start of the intervention, and a reminder list mid-intervention.

#### Skill-building and price reduction intervention

The combined group will receive (concurrently) both the skill-building and price reduction interventions detailed above.

#### Control group

Participants in the control group will complete the assessments only, until the intervention and six-month follow-up are complete, at which point they will be offered all print-based skill building intervention materials. Like the intervention participants, they will receive shopping vouchers and loyalty card points as compensation for their time and commitment (to the value of $75.00).

### Outcome assessments

Data will be collected pre- and immediately post intervention and at six-month follow up using a self-report questionnaire designed for the study which uses a combination of adapted previously validated measures and measures designed for the current study. The collection time-points for each measure are detailed in Table 2 below.

**Table 2:** Summary of study measures

Measures	Baseline	Post-intervention	Follow-up
Outcomes			
Vegetable purchasing and consumption	✓	✓	✓
Fruit purchasing and consumption	✓	✓	✓
Water purchasing and consumption	✓	✓	✓
Sugar sweetened beverage purchasing and consumption	✓	✓	✓
Shopping habits (location, frequency)	✓	✓	✓
Mediators			
Food security	✓	✓	✓
Self-efficacy for planning, shopping, preparing and eating fruit and vegetables	✓	✓	✓
Self-efficacy for drinking water and reducing sugar sweetened beverages	✓	✓	✓
Perceived affordability of fruit and vegetables	✓	✓	✓
Convenience	✓	✓	✓
Personal and household preferences	✓	✓	✓
Demographic and other covariates			
Age	✓	✓	✓
Height	✓	✓	✓
Weight	✓	✓	✓
Country of birth	✓	-	-
Relationship status	✓	-	-
Healthcare card holder	✓	-	-
Household composition	✓	-	-
Income (individual & household)	✓	-	-
Educational qualifications	✓	-	-
Employment status	✓	-	-
Whether currently pregnant	✓	✓	✓
Whether currently dieting	✓	✓	✓
Vegetarian status	✓	-	-
Smoking status	✓	-	-
Economic evaluation questions	✓	✓	✓
Process evaluation questions	-	✓	✓

#### Primary outcome measures

Purchasing and consumption of fruits, vegetables, and sugar-sweetened high-joule soft (carbonated) drinks vs. low-joule (carbonated) soft drinks/water are the primary outcomes. Data on purchasing of fruits, vegetables, high-joule sugar-sweetened soft drinks, low-joule soft drinks and water, and total grocery shopping purchases, will be gathered using electronic sales data, which Coles will provide for consenting participants via their store loyalty cards (Fly-Buys).

Fruit and vegetable consumption will be assessed using a series of self-reported food frequency questions adapted from the 1995 National Nutrition Survey which has been previously validated against food record data [36]. Measures of daily equivalent fruit and vegetable purchasing quantities (adjusted for household members/composition) and daily equivalent serves consumed will be calculated.

Sugar-sweetened high-joule beverage consumption, and low-joule beverage and water consumption will also be measured using a modified version of the previously validated measure [37] which asks respondents to record how much serves of each beverage they usually drink each day.

#### Mediator measures

Perceived barriers to fruit and vegetable consumption such as taste, availability, cost, quality, food waste, and knowledge about food preparation and meal planning will be assessed as potential mediators using items adapted from previously published scales [38-40]. Self-efficacy regarding reducing the consumption of sugar-sweetened high-joule beverages and increasing the consumption of water, and cooking skills will be measured using questions adapted from previously published measures [39,41]. Shopping practices and price considerations will also be measured as potential mediators using questions adapted from two previously published studies [22,42].

#### Demographics and other covariates

Demographic characteristics and other covariates will be assessed by questions on constructs as shown in Table 2, using standard measures.

#### Economic evaluation

A cost-consequences analysis will be conducted from a societal perspective comparing incremental costs and outcomes in each of the three intervention arms to the control arm. Main resource use data will be collected during the trial and follow-up period via surveys at baseline, pre- and post-intervention, project team records and Coles electronic sales data, supported by interviews with the project team. Household cost impact will be determined through external (Coles) data on food purchasing combined with individual self-report data on non-Coles and Coles-non-Flybuys food purchasing over the course of the intervention and follow-up period. Participant time and related travel expenses will be estimated from self-report data via study questionnaires. Sensitivity analyses will be conducted on key cost and outcome data. Given the short time-frame of the intervention and the follow-up period (less than one year), no discount factor will be applied to costs and outcomes in the analyses.

Economic evaluation results will give rigorous evidence to assist policy makers to make appropriate decision on resource allocation to such nutrition interventions at population level and to determine the cost-effectiveness of nationwide roll-out.

#### Process evaluation

Participants will be asked in the post-intervention and follow-up assessment to provide subjective evaluations of the usefulness of materials given and the discounts offered.

### Data analysis

Outcomes will be analysed on an intent-to-treat basis. Generalized Estimating Equations [43] will be used to fit regression models to describe the effects of the intervention on outcome and mediator variables. Generalized Estimating Equations represents an extension of the General Linear Model that allows parameter estimation for correlated data (e.g., from repeated measurements of participants). Models will be fitted to determine differences in changes in the outcome and mediator variables in the intervention and control groups. Potential confounders (e.g., age, education, country of birth, relationship status, employment status, household income, household composition) will be controlled for as necessary. Mediation analyses will be undertaken using the MacKinnon method [32]. Descriptive statistics (for quantitative data), content and thematic analysis (for qualitative data) will be used as appropriate to analyse the process evaluation data. StataSE v.11.2 [44] will be used for all statistical analyses.

## Discussion

The SHELf study is focused on developing evidence about effective means of improving fruit and vegetable consumption and decreasing intakes of sugar-sweetened beverages, behaviours that are established as key determinants of obesity risk [3,4,45]. It aligns directly with key national Health Priority Areas (e.g. as outlined at the 2008 Australian Health Minister's Conference, and in the recent Technical Report on Obesity in Australia [13], released by the National Preventative Health Taskforce).

The health, economic and social costs of poor nutrition are substantial. Historically the costs of nutrition-related disease in Australia were estimated at around $2.5 billion per annum [46]. As well as promoting and maintaining good health, sound nutrition confers substantial economic benefits that have impact across the community. For example, increasing fruit and vegetable consumption by just one serve per person per day in Australia would result in direct health care savings of $180 million/year [47] ($156.8 million/year savings for cardiovascular disease alone [48]).

An understanding of the effects of skills-based and price-related approaches to improving food purchasing and consumption behaviours will inform the evidence base upon which to build appropriate policy and program responses to the epidemic of obesity and poor nutrition currently facing Australia and other countries worldwide. For instance, results could provide support for skills-based programs that, being mail- or telephone-mediated, are relatively low-cost and could be implemented by governments or health care providers; and/or for tax-related or other fiscal policies aimed at shifting food purchasing behaviours at a population level.

There are also very few supermarket-based interventions reported internationally as such this is a major research gap and a potentially valuable missed opportunity in efforts to promote healthy eating behaviours. The link with Coles Supermarkets that this study would foster represents a rare and valuable public-private partnership opportunity to work collaboratively to promote healthy eating with a major national player in the Australian grocery retail sector. The research will contribute to informing the evidence base that is critically needed in order to reverse the epidemics of poor nutrition and associated adverse health outcomes, including obesity, in the Australian population and worldwide.

## List of abbreviations

SEIFA: Socioeconomic Index for Areas; SHELf: Supermarket Healthy Eating for Life

## Competing interests

The authors declare that they have no competing interests.

## Authors' contributions

KB conceived of and designed the study, developed the intervention and evaluation components, and drafted the manuscript. DC, SM, and CNM contributed to study design, intervention material development, and survey questionnaire design. VI contributed to study design, and oversaw Coles' contribution and logistics. NA contributed statistical and methodological expertise and generated the randomisation sequence. HL designed the economic evaluation. BM, DL contributed to documentation and refinement of study methods and manuscript preparation. CP contributed to study conception and intervention development. All authors read and approved the final manuscript.

## Pre-publication history

The pre-publication history for this paper can be accessed here:

http://www.biomedcentral.com/1471-2458/11/715/prepub
